# Asbestosis Mimicking Metastatic Lung Cancer: Case Report

**DOI:** 10.3390/medicina57050402

**Published:** 2021-04-21

**Authors:** Jin An, Minjeong Song, Boksoon Chang

**Affiliations:** 1 Department of Pulmonary, Allergy and Critical Care Medicine, Kyung Hee University Hospital at Gangdong, College of Medicine, Kyung Hee University, Seoul 05278, Korea; anjin7487@gmail.com; 2 Department of Pathology, Kyung Hee University Hospital at Gangdong, College of Medicine, Kyung Hee University, Seoul 05278, Korea; s10424@hanmail.net

**Keywords:** asbestosis, asbestos, lung nodules, lung cancer

## Abstract

The clinical diagnosis of asbestosis is primarily based on chest radiographic evidence of pleural thickening and interstitial fibrosis combined with a history of exposure to asbestos. We report herein the case of a 65-year-old man with asbestosis pathologically diagnosed after surgical lung biopsy. He had a work history including farming, cementing, and casting and was admitted with dyspnea. Chest computed tomography revealed multiple well-defined nodules in both lungs and a 4.1 cm peribronchial consolidation with fibrotic changes in the right lower lobe. We suspected metastatic lung cancer and video-assisted thoracoscopic biopsy was performed in the lung lesion of the right lower lobe. Asbestosis was confirmed following histological examination. The patient is currently completing outpatient visits without significant changes.

## 1. Introduction

Asbestosis is a fibrosing interstitial lung disease that can develop following occupational asbestos exposure and a lengthy latency period [1]. The clinical diagnosis of asbestosis is mostly based on chest radiographic evidence of interstitial fibrosis combined with a history of exposure to asbestos [2]. Chest radiography is important in the diagnosis, with key findings including pleural thickening and calcified pleural plaques, especially in chest computed tomography (CT); however, various chest CT findings of early to advanced asbestosis have been reported [3]. The characteristic chest radiographic findings of early asbestosis are small, irregular or reticular opacities, located predominantly in the lung bases [3]. Although lung biopsy is not usually necessary to make the clinical diagnosis when a significant exposure history is obtained, histopathology may be warranted to exclude lung cancer, which is the most common asbestos-induced neoplasm [1]. Here, we present an unusual case that presented as multiple lung nodules that mimicked metastatic lung cancer in chest CT and was diagnosed with asbestosis by surgical biopsy. 

## 2. Case Report

A 65-year-old man with a 20 pack-year history of smoking visited the emergency department with dyspnea for two days. The patient had several symptoms including cough and purulent sputum. He had worked in dairy farming for 20 years, cementing for 8 years, and casting for 9 years. In addition, he had participated in construction, replacing asbestos ceilings for 1 year at the age of 63 years without wearing a mask.

**Figure 1:**
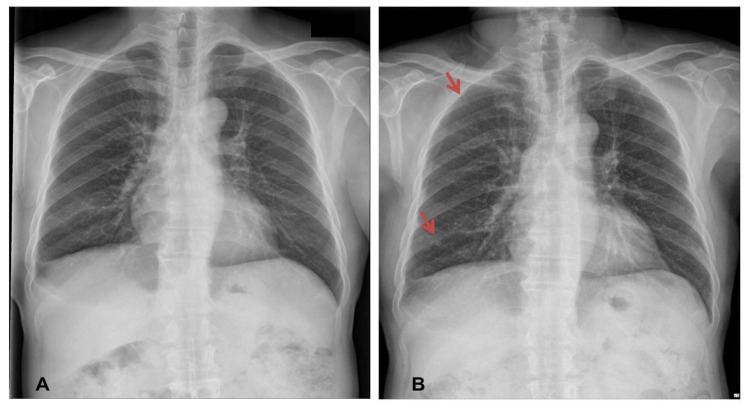
Comparison of chest X-ray obtained 12 years prior and on admission. Chest X-ray imaging revealed pleural thickening 12 years prior (**A**). Current chest X-ray showed a few nodular opacities with lung nodules and pleural thickening (**B**). The red arrows indicate lung nodules.

**Figure 2:**
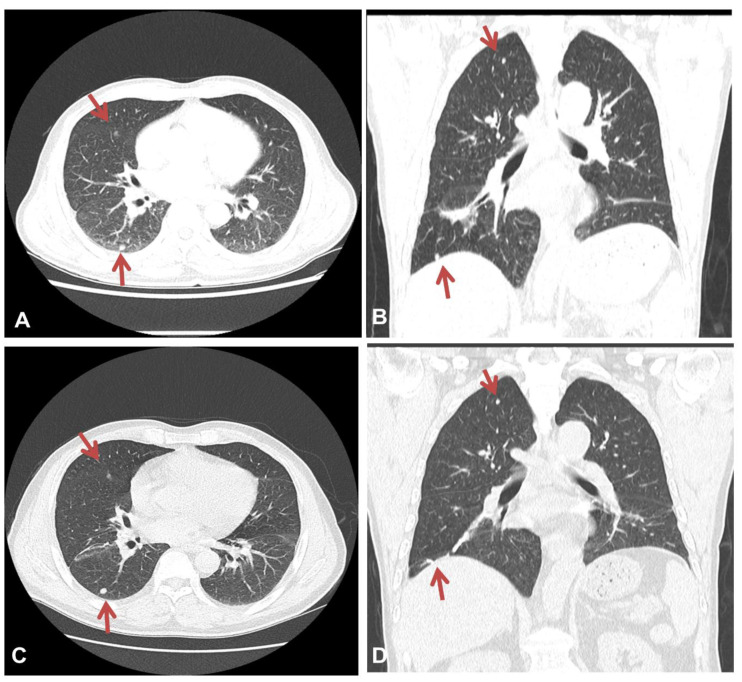
Chest CT findings at admission and 12 months after the diagnosis of asbestosis. Chest CT imaging revealed multiple well-defined nodules in both lungs (**A**) and a 4.1 cm peribronchial consolidation with fibrotic changes in the right lower lobe (**B**). There was no significant interval change in the multiple lung nodules on a chest CT scan taken 12 months after the diagnosis of asbestosis (**C**,**D**). The red arrows indicate lung nodules.

At admission, physical examinations showed no abnormal findings. The patient’s initial vital signs were as follows: blood pressure, 138/84 mmHg; body temperature, 36.3 °C; pulse rate, 92 beats/min; and respiratory rate, 20 times/min. The initial arterial blood gas analysis revealed a pH of 7.416, PaCO_2_ of 37.8 mmHg, PaO_2_ of 67 mmHg, and SaO_2_ of 93.0% (room air). Laboratory results revealed an increase in the serum levels of C-reactive protein (6.1 mg/dL), while other laboratory findings were within their normal limits. Chest X-ray imaging revealed features of bilateral nodular opacities with lung nodules and pleural thickening compared to a previous chest X-ray 12 years prior (Figure 1A,B). Enhanced chest CT showed multiple well-defined nodules in both lungs and a 4.1 cm peribronchial consolidation with fibrotic changes in the right lower lobe (Figure 2A,B). Empirical antibiotics (gemifloxacin 200 mg every 24 h) were introduced after bacterial cultures were obtained because bacterial bronchitis or organized pneumonia could not be ruled out based on symptoms of dyspnea, cough, and purulent sputum.

The pulmonary function test results were within normal limits; specifically, the forced expiratory volume in one second (FEV1) was 2.26 L (103% of the expected value), the forced volume vital capacity (FVC) was 3.01 L (99% of the expected value), and the patient’s FEV1/FVC was 75% of the expected value. His pulmonary diffusion capacity of carbon monoxide (DLco) was within the normal range at 13.8 mL/min/mmHg (92% of the expected value). Fiberoptic bronchoscopy did not reveal any significant endobronchial lesions. Bronchial washing for bacterial culture, acid-fast bacilli (AFB), tuberculosis polymerase chain reaction (PCR), and cytology were done in the right lower lobe, which showed consolidation on chest CT imaging; however, the results were all negative, and we excluded the possibility of pulmonary tuberculosis. Additionally, we excluded atypical pneumonia because serum *Aspergillus* antigen, *Cytomegalovirus* antigen, and *Cryptococcus* antigen tests were negative. Congestive heart failure was excluded by normal ultrasound cardiography results and the existence of a normal serum brain natriuretic peptide level. The patient’s dyspnea, cough, and sputum improved after administration of empirical antibiotics.

**Figure 3:**
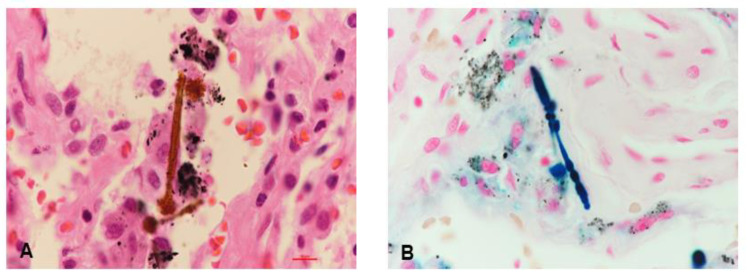
Asbestos bodies. Dumb-bell shaped ferruginous bodies, positive for iron staining, were identified in the alveolar space ((**A**), hematoxylin and eosin stain, ×1000; (**B**), iron, ×1000).

Although the levels of the tumor markers including carcinoembryonic antigen (CEA) and squamous cell carcinoma antigen (SCC) were normal, we suspected metastatic lung cancer due to the following: (1) the patient’s old age, (2) history of heavy smoking, and (3) well-defined multiple lung nodules on chest CT. Thus, video-assisted thoracoscopic (VATS) biopsy was performed in the peribronchial consolidation of the right lower lobe, and intra-alveolar asbestos bodies with pleural fibrosis were observed upon performing iron staining (Figure 3A,B). Finally, the patient was diagnosed with asbestosis caused by occupational asbestos exposure based on histopathological confirmation according to the clinical diagnosis criteria of asbestosis [4]. He was discharged with prescriptions for mucolytics and expectorants.

A follow-up chest CT scan performed at 3 and 12 months after the diagnosis of asbestosis showed no interval changes, with multiple well-defined nodules remaining apparent in both lungs (Figure 2C,D). The patient is currently undergoing outpatient visits with no significant changes.

## 3. Discussion

This is an uncommon case detailing the diagnosis of asbestosis that presents with multiple lung nodules that can be easily mistaken as metastatic lung cancer. Although pleural plaques on chest CT imaging are more frequently noted in asbestosis, an uncommon feature, i.e., multiple lung nodules, was observed in our patient. We initially planned surgical lung biopsy due to favorable findings for lung cancer on chest CT imaging, but asbestosis was ultimately confirmed with the observation of intra-alveolar asbestos bodies.

The current clinical diagnosis of asbestosis is mostly based on a history of exposure to asbestos combined with chest radiographic evidence of interstitial fibrosis and pleural plaques [2]. However, some patients are either unaware of or do not recall having been exposed to asbestos during previous work [2]. In our case, we did not initially obtain a detailed work history because the patient did not recall his various occupational histories. Typical radiologic findings such as interstitial fibrosis are similar to those of many other types of respiratory diseases. Asbestosis can also exhibit various atypical features such as parenchymal bands, ground-glass opacity (GGO), bronchiectasis, and honeycomb cysts [3]. A previous study found noncalcified nodules in 14% of asbestosis patients and asbestos-exposed workers [5]. Therefore, the current diagnostic criteria may be an inadequate approach to the diagnosis of asbestosis in some patients, as demonstrated by chest CT in our case. More specific diagnostic guideline needs to be presented for atypical cases of asbestosis. 

Clinical history is important for differentiating asbestosis and lung cancer. In patients with a history of asbestos exposure, asbestosis is the likely primary diagnosis, but lung cancer should be considered because asbestos is associated with lung cancer or malignant mesothelioma [6]. Lung cancer is also typically suspected in older patients with a history of heavy smoking; our patient had both of those characteristics as well as asbestos exposure history. Thus, clinical history related to asbestos exposure is an important clue in the initial differential diagnosis.

Multiple pulmonary nodules on chest CT are a sign of various benign inflammatory or infectious conditions, such as fungal infections, tuberculosis, and sarcoidosis. However, asbestosis is not generally considered a possible cause of multiple pulmonary nodules. In our patient, the presence of focusing lesion on chest CT (a consolidation in the right lower lobe) was more indicative of primary lung cancer than of distant metastasis from another primary malignant condition. However, primary lung cancer often spreads to the intrathoracic lymph nodes before spreading to other parts of the body including bilateral lungs. This finding without lymphadenopathy made a diagnosis of metastatic lung cancer uncertain. Bronchoscopic biopsy was not available because there were no endobronchial lesions or lymphadenopathy; thus, we planned surgical lung biopsy to ensure an accurate diagnosis. Presumably, the multiple lung nodules were anthracofibrotic nodules because no significant interval changes between the chest CT scans at admission and follow-up visits, respectively, were observed after the diagnosis of asbestosis. 

Establishing an accurate diagnosis is important for introducing earlier preventive therapy such as discontinuing asbestos exposure if ongoing and stopping smoking to improve the patient’s condition [7]. At the time of diagnosis, patients must be informed that they have a work-related illness that is progressive, increases their risk for malignancy, and results in respiratory failure [2]. Asbestos-related malignancies account for 3% to 8% of cases of lung cancers such as malignant mesothelioma [6]. Thus, active surgical biopsy may be necessary for an earlier diagnosis. Although a lung biopsy was performed for tissue analysis of asbestos fiber content in some previous cases [8,9,10], this was the first case with asbestosis that mimicked metastatic lung cancer and had asbestos bodies confirmed by surgical lung biopsy.

This case study was limited in terms of available information regarding the details of the patient’s work environment associated with exposure to asbestos. In particular, we did not assess the asbestos concentration and exposure period in his workplace. In addition, we did not perform ^18^F-fluorodeoxyglucose positron emission tomography/computed tomography (FDG PET/CT) because it is not currently covered by Korean national health insurance before histologic malignant confirmation and does not typically change the lung biopsy plan due to the potential overlap between benign and malignant conditions.

## 4. Conclusions

It is important to consider asbestosis in the case of unusual chest radiograph findings if the patient has a history of exposure to asbestos. Early surgical biopsy may be helpful to ensure accurate diagnosis and the timely introduction of preventive therapy including the discontinuation of both asbestos exposure and smoking. 

## Data Availability

The data reported in this paper are available from the medical history of the patient.
